# Regulatory agilities impacting review timelines for Pfizer/BioNTech’s BNT162b2 mRNA COVID-19 vaccine: a retrospective study

**DOI:** 10.3389/fmed.2023.1275817

**Published:** 2023-11-08

**Authors:** Prisha Patel, Judith C. Macdonald, Jayanthi Boobalan, Matthew Marsden, Ruben Rizzi, Marianne Zenon, Jinma Ren, Haitao Chu, Joseph C. Cappelleri, Satrajit Roychoudhury, Julie O’Brien, Konoha Izaki-Lee, Donna Boyce

**Affiliations:** ^1^International Regulatory Science and Policy, Pfizer, Tadworth, United Kingdom; ^2^International Regulatory Science and Policy, Pfizer, Kuala Lumpur, Malaysia; ^3^Global Regulatory Sciences, Pfizer, Tadworth, United Kingdom; ^4^BioNTech SE, Mainz, Germany; ^5^International Regulatory Science and Policy, Pfizer, Johannesburg, South Africa; ^6^Statistical Research and Data Science Center, Pfizer, Collegeville, PA, United States; ^7^Statistical Research and Data Science Center, Pfizer, Groton, CT, United States; ^8^International Regulatory Science and Policy, Pfizer, Dublin, Ireland; ^9^School of Physiology, Pharmacology and Neuroscience, University of Bristol, Bristol, United Kingdom; ^10^Global Regulatory Sciences, Pfizer, Collegeville, PA, United States

**Keywords:** regulatory, agilities, reliance, convergence, vaccines

## Abstract

The appropriate use of regulatory agilities has the potential to accelerate regulatory review, utilize resources more efficiently and deliver medicines and vaccines more rapidly, all without compromising quality, safety and efficacy. This was clearly demonstrated during the COVID-19 pandemic where regulators and industry rapidly adapted to ensure continued supply of existing critical medicines and review and approve new innovative medicines. In this retrospective study, we analyze the impact of regulatory agilities on the review and approval of Pfizer/BioNTech’s BNT162b2 mRNA COVID-19 Vaccine globally using regulatory approval data from 73 country/regional approvals. We report on the critical role of reliance and provide evidence that demonstrates reliance approaches and certain regulatory agilities reduced review times for the COVID-19 vaccine. These findings support the case for more widespread implementation of regulatory agilities and demonstrate the important role of such approaches to improve public health outcomes.

## Introduction

Regulatory agilities have been defined as the willingness of authorities to take quick action within the accepted regulatory framework, to ensure that the regulatory ecosystem swiftly responds to the challenges imposed by the pandemic for the ultimate benefit of patients and society as a whole (1). Some of these agilities existed pre-pandemic and some were established as a result of the COVID-19 pandemic. Irrespective, the application of regulatory agilities can be regarded as a hallmark of strong regulatory systems, enabling more efficient use of resources while safeguarding public health. Prior to the COVID-19 pandemic, many agencies already had tools/processes in place and employed procedures such as Emergency Use Authorizations (EUAs) sporadically and on a more restricted case-by-case basis with a limited impact to public health (2). The pandemic provided the impetus to maximize the usage and value of these pre-existing tools/processes, along with some additional agilities. This allowed the National Regulatory Agencies (NRAs) and Regional Regulatory Systems (RRS) to act quickly to ensure that patient access to both COVID-19 and non-COVID-19 medicines was enabled despite the challenges posed by the pandemic (3).

Previous publications have recommended improvements in regulatory efficiencies to drug development (4) and drug review process, including permanent adoptions of some of the agilities which were observed as a result of the COVID-19 pandemic (1, 5). These included greater international collaboration and reliance on other regulatory agency reviews and inspections, greater acceptance of digital tools and technologies for data capture and labeling as well as moving away from non-value-added requirements, such as provision of hard copy documents and individual batch release testing by regulatory agencies (5). These regulatory agilities enabled the timely registration and global distribution of life-saving anti-viral treatments and COVID-19 vaccines. These agilities should now be considered and leveraged to allow more streamlined registration of other important drugs and biologics. Reverting to the pre-pandemic regulatory processes is not in the best interest of public health and patients.

Accordingly, the time is right for establishing, where appropriate, and implementing agilities proven successful during the pandemic. These tools and processes will also establish best practices for responding to future emerging diseases. Both industry and regulators have cataloged the type of agilities employed in the pandemic (6–11). These were wide ranging in nature, for example, the International Federation of Pharmaceutical Manufacturers and Associations (IFPMA) defined three broad categories of agilities consisting of regulatory processes, clinical trials, and quality processes (6–8, 12).

In March 2020, Pfizer and BioNTech announced a collaboration to develop a COVID-19 vaccine. Initially, four nucleoside-modified messenger (mRNA)-based candidates were screened to identify the construct with the most promising safety and immunogenicity profile. This led to the selection of the modified mRNA BNT162b2^1^ construct for the Phase 2b/3 trial initiated in late July 2020 (13). Rolling review submissions to the United States Food and Drug Administration (US FDA) and European Medicines Agency (EMA) (14), as well as other agencies such as the UK Medicines Health Regulatory Authority (MHRA), started in early October 2020. This allowed those regulators early access to the dossier, and consequently prompt and progressive review of the data in real time, thus facilitating accelerated assessments.

**Figure 1:**
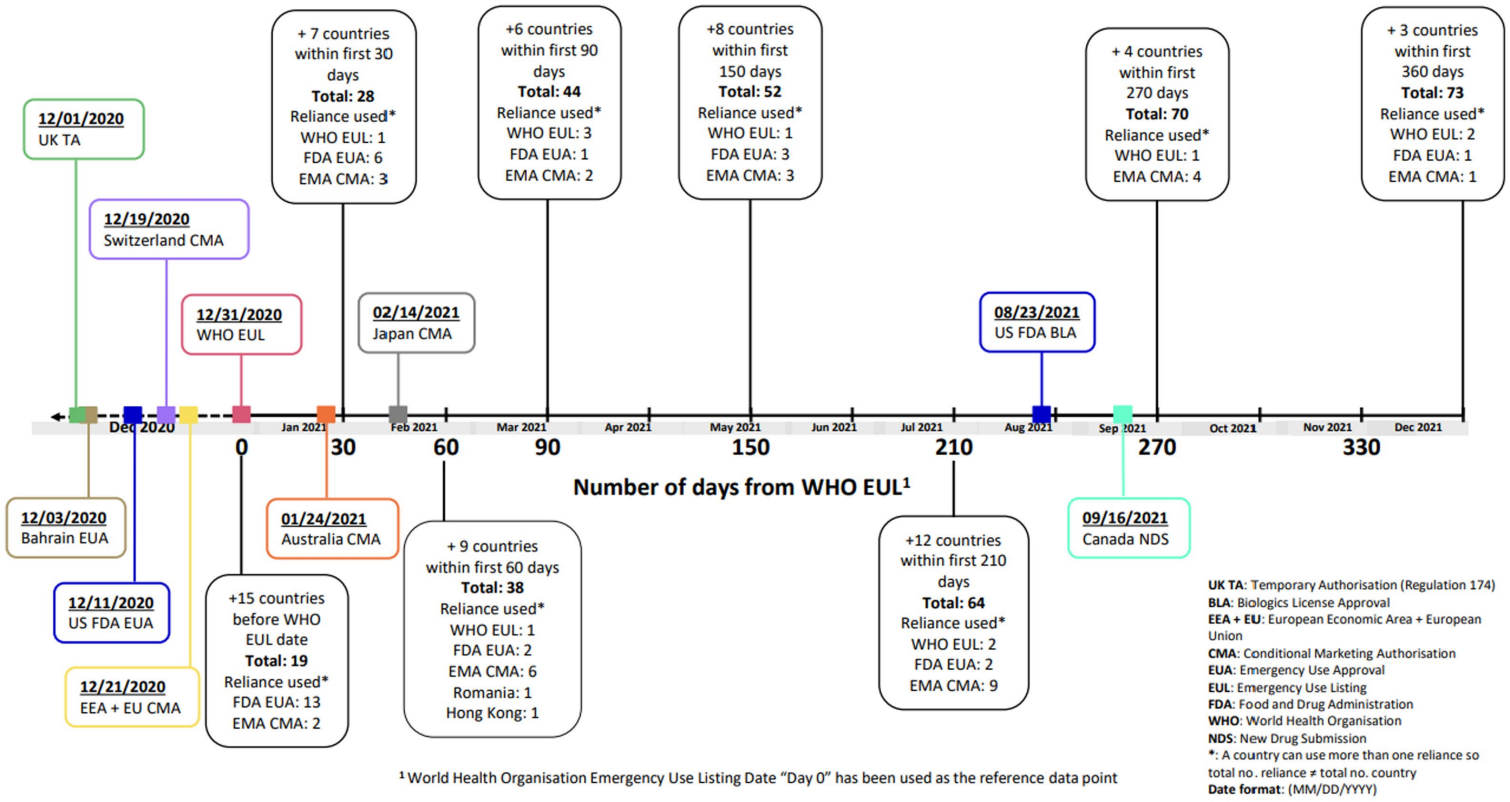
Timeline of regulatory approvals for BNT162b2 COVID-19 vaccine.

On 1st December 2020, the MHRA was the first regulatory agency to grant an authorization for the Pfizer-BioNTech vaccine, BNT162b2 mRNA COVID-19, under UK Regulation 174 (15). This was followed by the US FDA EUA approval on 11th December 2020 (16), the Swissmedic Conditional Marketing Authorization (CMA) on 19th December 2020 (17) and the European Union (EU) CMA on 21st December 2020 (18, 19). The World Health Organization (WHO) issued an Emergency Use Listing (EUL) on 31st December 2020, based on the EU CMA (20). Figure 1 illustrates the country approval times (following the WHO EUL) of BNT162b2 mRNA COVID-19 vaccine, using reliance pathways (i.e., the act whereby the NRA in one jurisdiction may take into account and give significant weight to assessments performed by another NRA or trusted institution, or to any other authoritative information in reaching its own decision). The relying authority remains independent, responsible and accountable regarding the decisions taken, even when it relies on the decisions and information of others (21, 22). The US FDA subsequently granted a Biologics License Approval (BLA) on 23rd August 2021, and the European Commission issued a full Marketing Authorization on 10th October 2022 for the COVID-19 vaccine (23, 24).

This paper will highlight the regulatory process agilities employed during the pandemic, especially those applied to the national requirements for marketing authorisations of BNT162b2 mRNA COVID-19 vaccine (Supplementary Table 1). Here we report on a distinctive retrospective study that examined whether the specific agilities granted had favorably impacted the regulatory review times for Pfizer/BioNTech BNT162b2 mRNA COVID-19 vaccine.

## Study objectives

There were two study objectives:

1. To compare the regulatory review times for BNT162b2 mRNA COVID-19 vaccine and Pfizer’s standard approval times (defined based on Pfizer’s historical records, using data for vaccines, New Biological Entities (NBE) and/or New Chemical Entities (NCE) case examples; noted in calendar days).
2. To investigate what types of regulatory agilities were employed by the relevant NRAs during review of BNT162b2 mRNA COVID-19 vaccine and determine if these agilities were associated with any change in the regulatory review times for BNT162b2 compared to Pfizer’s standard approval times for vaccines, NBE and/or NCE in the country. The regulatory agilities that were assessed are listed in Supplementary Table 1.

It is to be noted, hereafter, this paper will refer to BNT162b2 mRNA COVID-19 vaccine and Pfizer’s standard approval times for vaccines, NBE and/or NCE as COVID-19 vaccine and Pfizer’s standard approval time, respectively.

## Materials and methods

### Categorization of agilities

To identify the scope of the study, a cross-functional team was developed to determine which agilities (Supplementary Table 1) could be evaluated based on the observations made during review of the COVID-19 vaccine in the jurisdictions (1, 3, 25, 26).

### Data collection

Data were collected from Pfizer and BioNTech’s international Regulatory Affairs teams for 73 country approvals where COVID-19 vaccine has been approved by the NRAs or equivalent such as WHO by 31st March 2022.

Data were collected using Microsoft Excel for the identified data points from Pfizer’s and BioNTech’s internal database. BioNTech provided data for European Economic Area [EEA; + European Union (EU)], Hong Kong, Macau, Taiwan and Turkey; Pfizer provided data for the remaining countries.

To compare review times of COVID-19 vaccine against a standard reference point, data on Pfizer standard approval times (NCE, NBE and vaccines) were collected from Pfizer local countries’ internal databases.^2^ Any discrepancies in the data were clarified via discussion with the relevant point of contact responsible for the marketing authorization activities in the jurisdiction. This decision was taken because publicly available approval data metrics covering all the countries are not available.

### Study design and data analysis

This is a retrospective, observational study assessing the regulatory review times for COVID-19 vaccine compared with a standard baseline and assessing the impact of regulatory flexibilities on regulatory review times for COVID-19 Vaccine.

**Figure 2:**
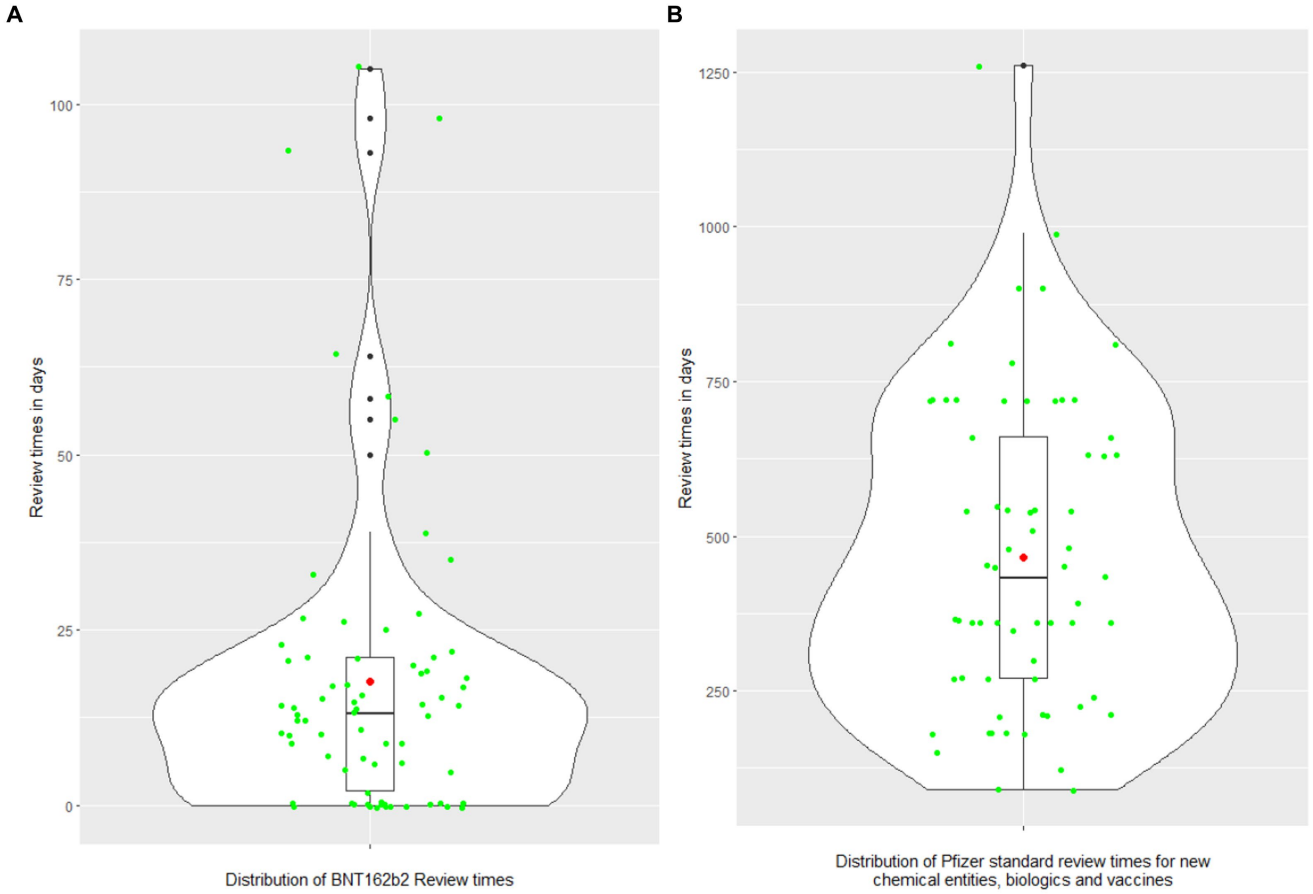
**(A,B)** The distributions of COVID-19 vaccine review times and Pfizer standard approval times. The violin plot includes a box and a kernel smoothed density plot. Box limits indicate the range of the central 50% of the data (i.e., the range between the 25th and 75th percentile), with a central black line marking the median value. The length of central vertical lines (also called whiskers) represents 1.5 times of interquartile range (i.e., the top of whisker is 1.5 times of 75th percentile), while the smoothed density lines represent the probability distribution. Jittered green dots represent the individual observations (countries). Black dots represent outliers and red dots represent the mean.

To address Objective 1 *“Describe the difference between the regulatory review times for COVID-19 vaccine and Pfizer’s standard approval times,”* descriptive analysis and violin plots (27) (Figures 2A,B), were used to display the distribution of Pfizer’s standard approval times and COVID-19 vaccine Marketing Authorization Application (MAA) review times for 73 jurisdictions, and the distribution of relative difference between these two types of review times. Each violin plot included a box plot where the box limits indicated the range of the central 50% of the data (i.e., the range between the 25th and 75th percentile) and the median value was marked by a central black line, and a kernel smoothed density plot representing the probability distribution was accompanied by Jittered green dots (individual observations).

The relative difference is presented in the paper^3^ and was defined as (Pfizer standard approval times-COVID-19 vaccine review times/Pfizer standard approval times × 100).

**Figure 3:**
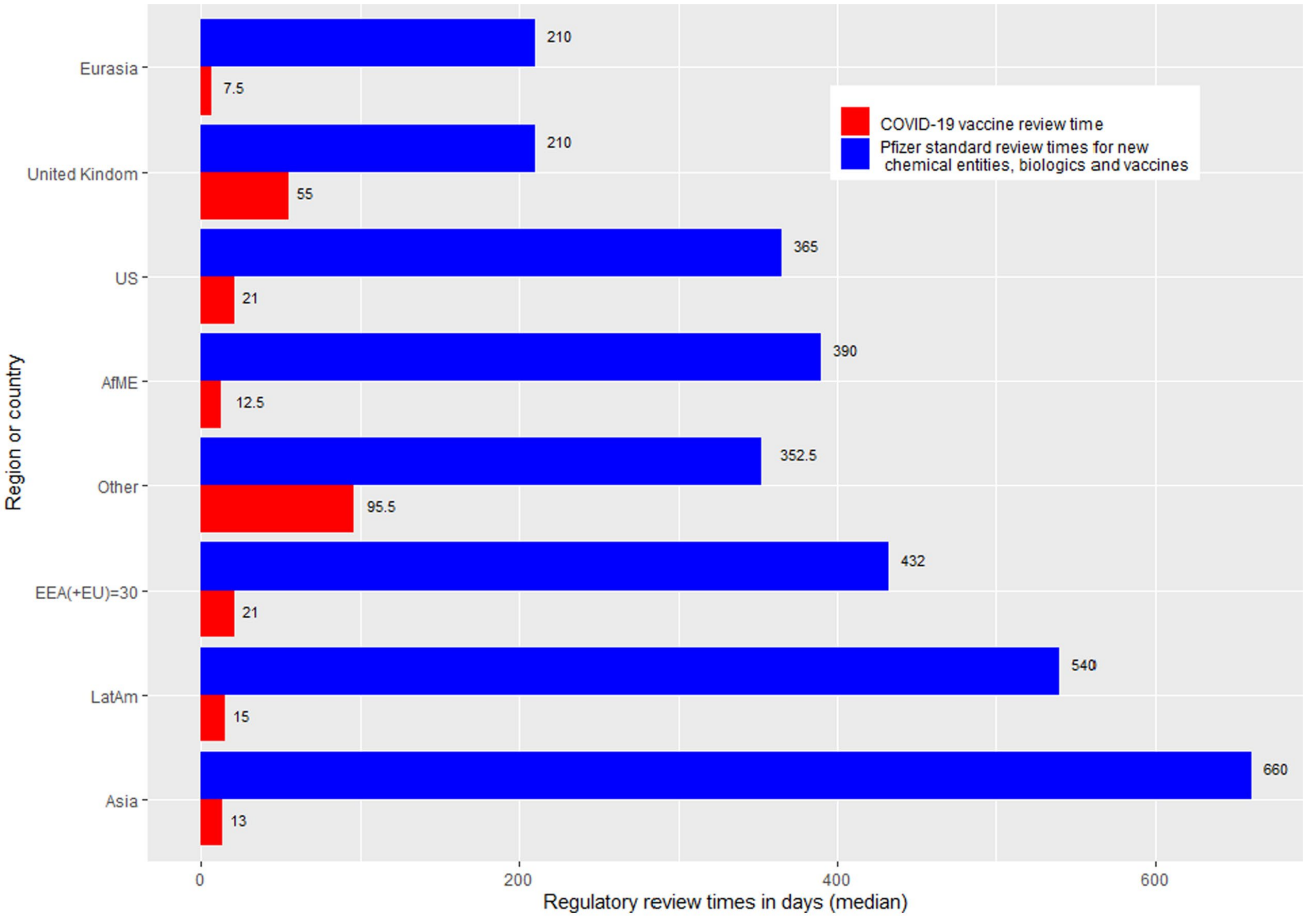
Bar chart showing regulatory review times by region. AfME, Africa and the Middle East; Other, Australia, Canada, Japan, and New Zealand; Asia, EEA, European economic area; Eurasia, Includes certain Europe and Asia countries that are not included in the other regional groups; LatAm, Latin America; UK, United Kingdom; US, United States of America.

The countries have been grouped in accordance with geographic and business considerations.^4^ The countries were grouped as Africa and Middle East, Asia, European Economic Area (EEA), Eurasia (includes certain Europe and Asia countries that are not included in the other regional groups), Latin America, United Kingdom, United States of America, and “Others” (Australia, Canada, Japan and New Zealand). Regional review time medians were calculated and plotted as a bar chart against Pfizer’s standard approval times. Individual country review times were overlaid on top of the stacked bar chart to further illustrate the difference of review (Figure 3).

The review times calculated for mRNA COVID-19 vaccine, to address Objective 1, included any of the agilities in Supplementary Table 1. To identify which specific agilities impacted the review times, further analysis on the agilities were conducted (Objective 2).

To address Objective 2 *“Investigate what types of regulatory agilities* (Supplementary Table 1) *were noted during review of COVID-19 vaccine and if these agilities are associated with the decrease of regulatory review times*,*”* both descriptive summary and statistical tests were conducted. The frequency and percentage of each type of regulatory agility used was calculated (Table 1). An agility was excluded from the frequency and percentage analysis if one or less agility was not utilized by any country (e.g., all countries selected “NO”), the categories had fewer than 10 observations, or more than 40% of observations with missing value (e.g., “not applicable,” “blank,” or “unknown”). Violin plots were used to display the distribution of percentage decrease in regulatory review times by each type of regulatory agility.

**Table 1:** Descriptive summary for regulatory agilities observed.

Question	Number of total responses	Number of “Yes” responses (% of “Yes response”)
Was reliance used for the review/approval?	73	62 (84.9)
Was the CPP* waived?	54	45 (83.3)
^Was local clinical trial and local clinical data waived?	ND	ND
Were agilities applied to the usual local CMC requirements (e.g., stability)?	61	60 (98.4)
Were local testing/lot release/sample requirements waived?	28	20 (71.4)
Were agilities applied to the Artwork/Labeling requirements?	71	68(95.8)
Were administrative/reference documents waived?	58	23 (39.7)
Were other agilities applied?	58	37 (63.8)

ND, No Data. ^*^CPP, Certificate of Pharmaceutical Product. ^**^CMC, Chemical Manufacturing and Control. ^^^Due to small sample size, analysis was not conducted. Swaziland—This market has no formal regulatory process and therefore was not included in the analysis. All waivers in the table relate to non-value-added national requirements that were duplicative of the core dossier.

Regression analysis (28) was conducted to identify if any type of agilities is correlated with the review times. Regression analysis was also conducted by region, where sufficient sample size was available. A quantile regression (29) was used for those variables that had outliers and extremely skewed distributions. These analyses is intended mainly for hypothesis generation and to provide a basis for future confirmatory assessment. Therefore, the *p*-values and 95% confidence intervals do not correspond to any prespecified set of hypotheses. The results regarding any inferences that could be drawn may not be reproducible and should be rather regarded as exploratory analyses generating hypotheses.

## Results

The results are presented in two parts: Part I—review times of COVID-19 vaccine compared to Pfizer’s standard approval times and Part II—impact of regulatory agilities (Supplementary Table 1) on review time.

### Part I—regulatory review time

Overall, regulatory approval time for the COVID-19 vaccine was significantly accelerated in all regions compared to the Pfizer standard approval times (*p* < 0.0001; Table 2; Figure 3). Supplementary Table 2 provides further breakdown and details of the review times for COVID-19 vaccine. The violin plot in Figure 2 shows the distribution of COVID-19 vaccine review times (Figure 2A) vs. Pfizer standard approval times for the countries (Figure 2B).

**Table 2:** Descriptive summary for regulatory review times (calendar days).

Type of regulatory review	Region^§^	*N*	Missing	Mean (SD)^#^	Median	Range	*p*-value^*^
COVID-19 vaccine review time	All regions	73	0	17.7(22.0)	13.0	0–105	<0.0001
	AfME	22	0	12(13.3)	12.5	0–50	
	Other	4	0	88.5(20.9)	95.5	58–105	
	Asia	16	0	14.4(10.9)	13.0	0–39	
	EEA (+EU) = 30	1	0	21.0(NA)	21.0	21–21	
	Eurasia	14	0	11.6(17.5)	7.5	0–64	
	LatAm	14	0	13.1(7.7)	15.0	0–26	
	United Kingdom	1	0	55.0(NA)	55.0	55–55	
	US EUA	1	0	21.0(NA)	21.0	21–21	
	US BLA	1	0	97.0 (NA)	97.0	97–97	
	WHO EUL^	1	NA	NA	10	10-10	
Pfizer standard approval times	All regions	73	6	478(253.6)	450.0	90–1,260	
	AfME	22	1	467.1(253.4)	390.0	150–990	
	Other	4	0	390(171.0)	352.5.0	225–630	
	Asia	16	4	673.1(233.9)	660.0	360–1,260	
	EEA (+EU) = 30	1	0	432.0(NA)	432.0	432–432	
	Eurasia	14	1	279.6(175.3)	210.0	90–720	
	LatAm	14	0	512.1(214.6)	540.0	120–810	
	United Kingdom	1	0	210.0(NA)	210.0	210–210	
	US BLA	1	0	365.0(NA)	365.0	365–365	

^§^Region: AfME (Africa and the Middle East), Other (Australia, Canada, Japan, and New Zealand), Asia, EEA (European Economic Area), Eurasia (includes certain Europe and Asia countries that are not included in the other regional groups), LatAm (Latin America), UK (United Kingdom), and US (United States of America). N, number of countries in the regional cohort. It is to be noted that the total number of New Chemical Entities (NCE), New Biological Entities (NBE), and vaccines used to determine Pfizer standard approval times for each country has not been provided. ^^^WHO EUL (World Health Authorization Emergency Use Listing). ^*^Paired *t*-test was conducted to examine the difference between COVID-19 vaccine review time vs. Pfizer standard approval times for all regions. Statistical tests were not conducted for each region due to the small size. ^#^SD, standard deviation; NA, not available.

Of the 73 countries included in the analysis, one required full approval (Israel), one was approved via New Drug Submission-COVID-19 process (Canada), six required only an Import License/Permit to make the vaccine available, 49 were approved via EUA and the remaining 16 countries allowed for approval via CMA (Supplementary Table 2). It was noted 52 of the 65 countries did not have any EUA or CMA procedure in place prior to the pandemic. There were six countries where data on Pfizer standard approval time data were not provided (either unavailable or unknown).

Many NRAs relied on the WHO EUL (based on the EU CMA) which was granted on 31st December 2020 (Figure 1). The EMA facilitated sharing the data and EMA assessment reports with WHO, as well as the WHO participating in EMA meetings (30). This allowed other countries to use WHO EUL to facilitate reviews and decision making for their jurisdiction. Further regional observations are detailed in Table 3.

**Table 3:** Descriptive summary of regional regulatory observation for COVID-19 vaccine.

Region	Regulatory observations
Africa Middle East (AfME)	The types of waivers/agilities granted were country-dependent and based on whether a specific waiver request was made for a particular requirement or not. Majority of the countries had no formal EUA processes and little or no guidance available from the outset. 9 countries relied solely on WHO EUL to grant approval, without the need for additional in-country submissions and review. Bahrain was the second market globally to receive an EUA approval two calendar days after the UK. Algeria granted a waiver from the mandatory pre-step requirement that usually requires pre-submission step before a regulatory submission can proceed. This step requires the applicant to provide an overview of the medical and therapeutic value of the product, in addition to proposing pricing and detailing how the medicine will improve public health in Algeria. Not only were there agilities applied in the review processes, but there were agilities observed in terms of the submission requirements that are mandatory under usual regulatory review. Agilities or waivers were granted, for the provision of Certificate of Pharmaceutical Products (CPPs), product samples, pricing certificates, provision of full Common Technical Documents (CTD) and country-specific artworks.
Others (Australia, Canada, Japan, and New Zealand)	3 out of these 4 countries were Stringent Reference Authorities (31) (Australia, Japan, and Canada). Figure 3 and Table 2 show this cohort of countries had the longest median approval time (95.5 calendar days) for COVID-19 vaccine, compared to the other regions. This may be due to the type of review the cohort of agencies conducted, such as an independent review.
Asia	Bhutan, Brunei, Maldives accepted administrative documents and did not require a registration dossier submission. Macao and Hong Kong required only import license applications. 10 countries obtained approval in less than a month. Vietnam, which has one of the longest approval times from Pfizer’s standard review time, took 11.0 calendar days to approval via the EUA pathway.
Eurasia	Armenia, Moldova, Georgia, Mongolia, Palestine, Turkey did not require dossier submission. Switzerland did not have emergency use regulation and conducted a rolling review.
European Economic Area (EEA) Observations	EMA’s rolling review of the data was started prior to the official clock start, while the formal assessment was initiated once EMA deemed that a sufficient body of data had been submitted during the rolling review period, thus facilitating the expedited formal review time. CMA was granted in the EU 21.0 calendar days following the official clock start.
Latin America (LatAM)	Several NRAs introduced a variety of approaches to ensure timely authorization of COVID-19 therapies, including the establishment of legislation to support timely authorization of the vaccines and other therapies. It was observed that several post-approval changes were implemented following notification from the Marketing Authorization Holder (MAH) in Argentina. Brazil issued specific changes to the legislation (32), allowing the vaccine full registration within 1 month (18.0 calendar days) (Supplementary Table 2). Brazil granted waivers to provide Phase 3 pending study results as part of a post approval commitment. Mexico issued special regulations (33) with reduced approval times and other agilities following alignment with EU CMA and FDA’s EUA.
United Kingdom	Temporary authorisation under Reg.174 (15) was given within 55.0 calendar days, using an expedited rolling review (Supplementary Table 2).
USA	Approval was received within 21.0 calendar days via the EUA procedure. Though the EUA is not directly comparable to the BLA, due to the reduced data requirements; it is to be noted that US FDA started their rolling review of the data in advance of the formal EUA procedure. The real-time data provision facilitated the rapid review cycle for the EUA and similarly subsequently paved the way for the rapid review time of 97.0 calendar days for the full BLA (Table 2).

### Results part II—impact of regulatory agilities on review time

Review times are strongly influenced by reliance (34–36). Therefore, a question on reliance utilization: “Was reliance used in the review/approval process” was posed to the Pfizer staff in 73 countries (Table 1). Sixty-two out of seventy-three (84.9%) countries noted that reliance was used for the review/approval of COVID-19 vaccine. Other agilities that were observed are shown in Table 1.^5^ The most widely used agilities were noted for Chemical Manufacturing and Control (CMC) requirements (98.4%, *n* = 61), labeling (95.8%, *n* = 71) and waiving of Certificate of Pharmaceutical Product (CPP) requirements (83.3%, *n* = 54).

**Figure 4:**
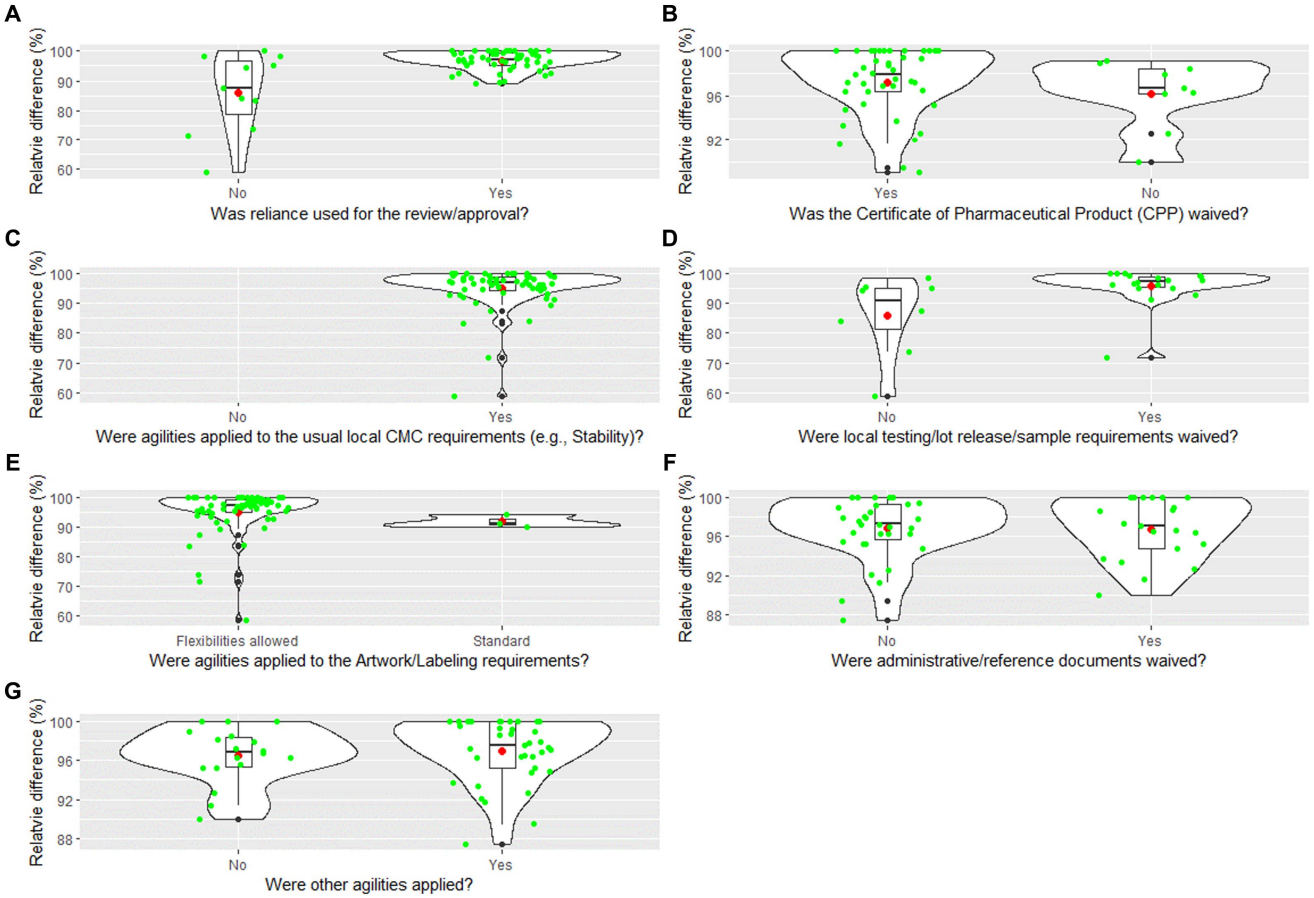
**(A–G)** Violin plot for the relative difference between COVID-19 vaccine review times and Pfizer standard approval times by regulatory agilities. The violin plot includes a box and a kernel smoothed density plot. Box limits indicate the range of the central 50% of the data (i.e., the range between the 25th and 75th percentile), with a central black line marking the median value. The length of central vertical lines (also called whiskers) represents 1.5 times of interquartile range (i.e., the top of whisker is 1.5 times of 75th percentile), while the smoothed density lines represent the probability distribution. Jittered green dots represent the individual observations (countries). Black dots represent outliers and red dots represent the mean.

Figures 4A–G shows the distribution of the responses, as violin plots for specific regulatory agilities applied and the relative difference between COVID-19 review times and Pfizer standard approval times:

- Was reliance used for the review/approval?
- Was the Certificate of Pharmaceutical Product (CPP) waived?
- Were agilities applied to the usual local CMC requirements (e.g., Stability)?
- Were local testing/lot release/sample requirements waived?
- Were agilities applied to the Artwork/Labeling requirements?
- Were administrative/reference documents waived?
- Were other agilities applied?

As a way to enable a confirmatory analysis, regression analysis was performed to determine if there was a correlation between the agilities granted (Supplementary Table 1) and reduction in review timeline for COVID-19 vaccine.^6^ This analysis (Table 4) demonstrated 2 agilities may contribute to a reduction in review timelines. The two agilities noted were use of reliance in the review/approval process and agilities allowed for artwork/labeling requirements.^7^

**Table 4:** Regression analysis to determine correlation between use of regulatory agilities and the decrease of regulatory review time.

Question (Yes vs. No)	Estimate of relative difference (%)^#^	95% CI
Was reliance used in the review/approval? (Yes)	10.97	(7.16, 14.79)^***^
Subgroup in Asia	−1.08	(−4.57, 2.42)
Subgroup in Europe	2.50	(−4.70, 9.69)
Was the CPP waived? (Yes)	0.96	(−1.22, 3.15)
Subgroup in Asia	0.80	(−1.00, 2.59)
Subgroup in Eurasia	6.28	(0.60, 11.97)
^Was local clinical trial and local clinical data waived?	ND	ND
^§^Were local testing/lot release requirements waived? (Yes)	2.70	(−8.88, 14.27)
Subgroup in Eurasia	11.72	(1.26, 22.17)
^§^Were agilities applied to the Artwork/Labeling requirements? (Yes)	5.89	(1.90, 9.88)^**^
^Were agilities applied to the usual local CMC requirements (e.g., stability)?	ND	ND
Were administrative/reference documents waived (Yes)	−0.07	(−1.77, 1.63)
Subgroup in Eurasia	0.81	(−5.38, 7.00)
Were other agilities applied (Yes)	0.46	(−1.30, 2.22)
Subgroup in Eurasia	0.91	(−4.82, 6.62)

^*^p < 0.05. ^**^p < 0.01. ^***^p < 0.001. CI, confidence interval; CPP, certificate of pharmaceutical product; CMC, chemical manufacturing and control; ND, No Data (or no sufficient data for regression analysis). ^^^Due to small sample size, analysis was not conducted. Subgroup analysis was conducted only if the sample size was available for the specific region. ^§^A quantile regression was used for those variables that had outliers and extremely skewed distributions. ^#^The relative difference was defined as (Pfizer standard approval times – COVID-19 vaccine review times/Pfizer standard approval times × 100). For instance, the estimate of 10.97 indicates reliance used (yes) in the review/approval decreased the regular review time by 10.97%, but the estimates were varied in the subgroup analyses (−1.08% in Asia and 2.5% in Europe).

The use of reliance in the review/approval decreased the regulatory review time by 10.97% (95% CI 7.16–14.79%, *p* < 0.001) and agilities in labeling requirement decreased the review times by 5.89% (95% CI 1.90–9.88%, *p* < 0.01).

Regional analysis was conducted to determine if there were any differences and significance between the agilities granted with respect to approval times (Table 4). The regression analysis determined that no agilities contributed to a statistically significant reduction in review times by region. In addition, it was observed that most countries accepted the English Language Dossier (including countries where a local language or dual language dossier was required) with the exception of Japan, South Korea, and Taiwan.

## Discussion

This study provides evidence that reliance approaches and agilities reduced review times for the COVID-19 vaccine (Figure 2). While this finding is not surprising, especially in the globally galvanizing context of a shared public health emergency, the data also reveal the specific agilities which had the greatest impact in reducing approval times. The most dramatic effects were seen in two areas: labeling and CMC requirements agilities. The use of labeling agilities was statistically significant in terms of a correlation to review time via regression analysis (Table 4), were widely applied (95.8%; Table 1), and impactful on approval times (Figure 4E).

Although agilities in provision of additional national CMC requirements to core Common Technical Documents (CTD) sections were not statistically significant in the regression analysis, they were also widely applied (98.4%; Table 1), and the positive impact on approval times is clear (Figure 4C). Another CMC related area that is problematic for industry is duplicative import testing in countries when results from either the manufacturer or another regulator are available. Our study showed that this was waived in 71.4% of countries, however, this agility failed to show statistical significance in the regression analysis, although Figure 4D shows that it contributed to the improvement in review times observed. Nonetheless it remains a significant pain point for the countries (37–39).

### Agilities that could be applied outside of a pandemic setting

There have been numerous publications addressing which of the agilities applied during the pandemic could be carried forward as standard practice (1, 4, 10, 12, 25, 40–42). In the opinion of the authors, this study makes a strong case for carrying forward labeling agilities. These agilities consisted of the acceptance of artwork and labeling for common English only packaging, instead of providing country specific/local language packaging, thus facilitating speed and ease of distribution across countries/regions. The acceptance of packaging QR codes instead of physical labels, also allowed both easy access to the most current version and for healthcare providers to view local language product information to ensure correct usage and administration of the vaccine. The pain points of the usual provision and updating of paper patient leaflets and summary of product characteristics outside of the pandemic have been well documented (26) and many regulators are embracing e-labeling (25) as these formats can advance health literacy and adherence. We believe that this progress together with the experience in the pandemic, should accelerate the acceptance of e-labeling. However, implementation may be more challenging in low-middle-income countries which may have sub-optimal availability of technologies and therefore hinder digitalization of working practices (7). Similarly, there is arguably no place for non-value add national CMC requirements since the COVID-19 approval experience has proven that these were not needed for risk/benefit analysis.

### Harmonization and convergence are key to reliance

The future state envisaged cannot be achieved without the key enablers of harmonization of technical requirements and convergence of review practices. The International Council of Harmonization for Technical Requirements for Pharmaceuticals for Human Use (ICH) plays a crucial role in the harmonization of technical requirements to provide regulatory relief from conflicting requirements that slow development and continues to make vital progress in this area. Nevertheless, convergence of regulatory review practices can be more challenging. For example, there is evidence that having a common CMC data set at submission does not guarantee a harmonized pharmaceutical control strategy as regulators have differing review practices, leading to divergence during assessment (33).

Throughout the pandemic, the International Coalition of Medicines Regulatory Authorities (ICMRA) had a pivotal role in supporting strategic coordination and international cooperation among global medicine regulatory authorities (43). In a very positive move, ICMRA is currently conducting a collaborative pilot with industry on pharmaceutical quality knowledge management systems (44). They state that “A better understanding of areas of potential alignment and difference is an important first step to harmonizing specific CMC and inspection-related regulatory procedures to facilitate the timely implementation of appropriate regulatory actions across different regions” (44).

### Future state—where are we heading?

In terms of rolling review, regulators are understandably skeptical about the feasibility of its widespread application to the review of medicines outside of a pandemic setting due to resource considerations. Nonetheless, the COVID-19 pandemic experiences have disrupted and challenged the usual regulatory review paradigm of submitting all the required clinical, manufacturing, and labeling data in a single large MAA with no earlier review of components. Considering the regulatory flexibility demonstrated during the pandemic, it is now timely to challenge the accepted wisdom of this approach. There is a growing consensus that the submission and review of regulatory documentation needs to be freed from the shackles of “electronic paper” which traps data in making it hard to analyze, and update formats (26). Hence, it is reasonable and appropriate to move toward an earlier and more iterative exchange of clinical, non-clinical and manufacturing data intended to support drug or vaccine registrations as the data is generated, reflecting the continuous generation of knowledge across the research and development cycle, offering the possibility for a more seamless knowledge management. The European Federation of Pharmaceutical Industries and Associations (EFPIA) has developed the concept of “Dynamic Regulatory Assessment” (45, 46) that envisages the provision of agreed discrete data packets at timepoints in advance of the full MAA (47, 48). This is more of a phased review and can be considered as a first step toward evolving the review process in the future. Further innovations in this field are in play to move toward structured data that are both human and machine readable, enabling efficiencies from automation and the housing of data in a multi-tenant, multi-residency cloud-based platform (47, 48).

To complement the agilities afforded by global regulators and to further aid and expedite the development and registration of breakthrough medicines and vaccines at Pfizer, we focus on what we term “lightspeed” programs and a lightspeed culture. These actions were further honed during our experience of developing the COVID-19 vaccine with our partner BioNTech, where as soon as data were generated, they were shared with regulatory agencies as they became available, resulting in unprecedented development and approval times globally. Now established, these successful actions have challenged and changed expectations for other serious and life-threatening diseases (49). As a result, the Pfizer approach to global regulatory sciences has evolved and the “lightspeed mentality” is now applied to all global development programs. Similarly, other drug developers are shifting toward a more iterative and agile approach to regulatory science, and regulators are actively involved in discussions on long-term implementation of more dynamic frameworks for pandemic preparedness and beyond.

### Limitations

Despite the clear impacts shown in the use of reliance and associated agilities, there are a number of limitations of our study. For example, data were not collected on the use of rolling review practices across countries, although this practice was commonly applied in early approvals from stringent regulatory agencies (e.g., EEA, USA, Canada, and UK). It would have been useful to explore this in more detail. Furthermore, data were categorized into regional groupings that reflected Pfizer’s organizational structure and not necessarily regulators of similar philosophy and resourcing level. This made it difficult to evaluate regional trends, however it was recognized that some geographic regions are intrinsically very diverse in their own right (e.g., Asia).

## Conclusion

This study demonstrated which agilities impacted the regulatory review times in a pandemic scenario and showed that many of these agilities did lead to reduced review times for the COVID-19 vaccine as compared to the Pfizer standard approval times. The WHO EUL was a significant milestone and this was facilitated by WHO observing the EMA review, further our approach of registering all manufacturing sites under a single EUL simplified subsequent updates and facilitated the rapid roll-out of the vaccine affording flexibility of supply to Lower- and Middle-Income Countries. It can be inferred that the WHO EUL approval was a pivotal moment judging by the clustering of numerous approvals which followed this (Figure 1).

The lessons learned from this study could support streamlining not only future pandemic regulatory processes, but also open opportunities for some best practices to be adopted into routine review and considerations for strengthening the regulatory system/environment.

There are also further areas that could be explored in future publications such as the use of expedited pathways, that is, whether regulators had mechanisms in place to expedite approvals and how these pathways were used during the pandemic. Another area of interest could be the management of post approval changes, that is, did the agilities applied pre-approval lead to an increased workload of CMC post approval changes and how is that being handled? These are all areas which could be investigated in the future.

An area which our study did not address was the use of real-world evidence (RWE) and the use of platform approaches. There is a growing acceptance by regulatory authorities of using RWE to support regulatory review. For the COVID-19 vaccine and Pfizer’s anti-viral product, RWE was key to support EUAs and to support registration for the current indications and dosing regimens. RWE will continue to be leveraged to support additional labels claims for the COVID-19 product portfolio including potentially prevention against “long covid” symptoms. Beyond COVID-19 vaccines and COVID-19 antivirals, prospective RWE studies are planned to support new label claims, pre and post registration, across the Pfizer portfolio from oncology to internal medicine drugs.

There is also a growing acceptance for “Platform Approaches” that were also instrumental in the context of COVID-19 vaccines development. The mRNA platform was utilized to develop the COVID-19 vaccine which, following initial complete non-clinical/toxicological work, was studied in more than 40,000 adults in the clinical development program and subsequently administered to millions of individuals globally since it was first authorized in 2020.

The experience gained by both Pfizer/BioNTech and regulatory authorities with the original COVID-19 vaccine using this mRNA platform enabled the rapid development of the Omicron-adapted bivalent variant vaccines. This meant clinical data from the original BNT162b2 vaccine, as well as multiple BNT162b2-based variant-adapted vaccine candidates, could be leveraged to support an expedited approval of the bivalent Original / Omicron BA.4–5 vaccine without requiring repetition of toxicological work and before clinical trial data on the specific vaccine were available. The same approach based on the well-established mRNA-LNP platform will continue to support the accelerated development and registration of new COVID-19 variant vaccines and it is being utilized to rapidly develop vaccines against other viral infections including influenza and varicella. With the regulatory agency acceptance of “platform data,” these new mRNA-based vaccines are progressing through clinical development efficiently enabling more timely registration and an overall reduction in the amount of clinical and manufacturing data required prior to registration.

To conclude, this study, to our knowledge the first of its kind, has demonstrated that the adoption of regulatory agilities in the face of the COVID-19 pandemic was transformational in speeding the delivery of public health solutions. Adoption of regulatory agilities have the potential to be similarly impactful for the future regulatory environment.

## Data availability statement

The datasets presented in this article are not readily available because they contain raw data that contain proprietary information and survey results on additional research aspects that may be used to support further publications. Upon request, and subject to review, Pfizer may provide the data that support the findings of this study. Requests to access the datasets should be directed to the corresponding author.

## Author contributions

PP: Conceptualization, Data curation, Methodology, Project administration, Validation, Writing – original draft, Writing – review & editing. JM: Writing – original draft, Writing – review & editing. JB: Conceptualization, Data curation, Methodology, Writing – original draft, Writing – review & editing. MM: Writing – original draft, Writing – review & editing. RR: Writing – review & editing. MZ: Conceptualization, Data curation, Methodology, Writing – original draft, Writing – review & editing. JR: Formal analysis, Visualization, Writing – review & editing. HC: Formal analysis, Visualization, Writing – review & editing. JC: Formal analysis, Visualization, Writing – review & editing. SR: Formal analysis, Visualization, Writing – review & editing. JO’B: Conceptualization, Methodology, Writing – original draft, Writing – review & editing. KI-L: Data curation, Methodology, Writing – review & editing. DB: Writing – review & editing.

## Footnotes

^1^Modified mRNA—BNT162b2 is a lipid nanoparticle-formulated, nucleoside-modified mRNA vaccine for the prevention of the novel coronavirus disease 2019 (COVID-19) caused by severe acute respiratory syndrome coronavirus 2 (SARS-CoV-2) infection.

^2^Africa, Asia, Eurasia, Latin America, Middle East, and New Zealand, the Pfizer standard approval times for each country are derived from previous Pfizer approval experiences (new chemical entity, biologics, and vaccines). Where external information is available, such as other company experience or the regulatory agency has published actual review times data, these data are also considered to determine the approval time. Where agencies have published target times, these are only considered if they are representative of Pfizer’s or other companies experience. Pfizer’s standard approval times are reviewed annually, to ensure they are comparative to the regulatory environment in that country or aligned to Pfizer’s experience. European Economic Area [EEA; + European Union (EU)], Australia, Canada, Japan, United Kingdom and United States of America, the agency target times are used as the reference for Pfizer standard approval times (United Kingdom baseline reference approval times are representative of the target timelines when the data was provided for this study and may not be reflective of the current process).

^3^The raw difference was also calculated; however, due to the considerable variation of Pfizer standard approval times across countries, the relative difference was used.

^4^Africa and Middle East Region: Algeria, Bahrain, Botswana, Egypt, eSwatini (Swaziland), Ghana, Iran, Iraq, Jordan, Kenya, Kuwait, Lebanon, Mauritius, Morocco, Nigeria, Oman, Qatar, Rwanda, Saudi Arabia, South Africa, Tunisia and United Arab Emirates. Other: Australia, Canada, Japan and New Zealand. Asia: Bhutan, Brunei, Hong Kong, Indonesia, Macao, Malaysia, Maldives, Nepal, Pakistan, Philippines, Singapore, South Korea, Sri Lanka, Taiwan, Thailand, and Vietnam. EEA, Eurasia (include Europe and Asia countries that are not included in the other category groups): Albania, Armenia, Georgia, Israel, Kazakhstan, Kosovo, Moldova, Mongolia, North Macedonia, Palestine, Serbia, Switzerland, Turkey and Ukraine. Latin America: Argentina, Brazil, Chile, Colombia, Costa Rica, Dominican Republic, Ecuador, El Salvador, Honduras, Mexico, Panama, Paraguay, Peru and Uruguay. United Kingdom and United States of America.

^5^Questions on agilities being applied to local clinical and non-clinical requirements were requested. However due to the sample size, violin plot and regression analysis were not conducted.

^6^Regression analysis was not conducted on local Chemical Manufacturing and Control (CMC) requirements as there was only one country response “No” to the question “were agilities applied in the usual local CMC requirements (e.g., Stability)?”

^7^The use of reliance was selected if the NRA observed the acceptance of the SRA’s benefit-risk decision in approving the product and may include exemptions of country specific/local documents (i.e., documents that do not form part of a routine registration requirement per International Conference of Harmonization (ICH) standards and is specific to a country requirement) across all modules. Artwork/labeling agilities were selected if one or more of the following were observed: Exemption of country specific details/artwork; Acceptance of common English labels; Exemption of including physical leaflets on product packs and/or acceptance of QR code on pack (to access local labels) in replacement of physical local labels (Supplementary Table 1).
