# Supplementary material for: Regulatory agilities impacting review timelines for Pfizer/BioNTech’s BNT162b2 mRNA COVID-19 vaccine: a retrospective study
Source: Front Med (Lausanne). 2023 Nov 8;10:1275817. doi: 10.3389/fmed.2023.1275817 (PMC10664654; doi:10.3389/fmed.2023.1275817)
Supplement: Supplementary file 1 [file Table_1.DOCX]

Supplementary Table 1. Regulatory agilities used to assess the impact on review times

| **Regulatory Agilities** | **Description** |
| --- | --- |
| Reliance used in the review/approval | Acceptance of the NRA to reference a SRA’s benefit- risk decision in approving the product and may include exemptions of Country specific/local documents (documents that do not form part of a routine registration requirement per International Conference of Harmonization (ICH) standards and is specific to a country requirement) across all modules |
| Certificate of Pharmaceutical Product (CPP) | CPP related exemptions which may include any of the following:   1. Agreement to provide CPP during review instead of prior to or at submission. 2. Acceptance of alternate documents to CPP 3. Waiver for legalization/notarization requirements |
| Local clinical trial and local clinical data | Exemption of requirements/document (national/local requirements) related to Module 4 (non-clinical) and/or Module 5 (Clinical) documents (as applicable) |
| Chemical Manufacturing & Control (CMC) | Flexibilities related to Core ICH content included in Module 2.3 or 3.2 S/P of registration dossier which may include any of the following:   1. Allowance for rolling submission, options to provide minimal data to be supplemented with full data during review/upon availability, etc. 2. Exemption from provision of product, or site-specific document (country specific requirements) in addition to the core ICH content included in Module 2.3 or 3.2 S/P of registration dossier) 3. Batch documents flexibilities |
| Local testing/lot release/Sample | Exemption of duplicative Lot Release/Local testing requirements which may include exemption of need to provide sample for testing or for physical reference purposes |
| Artwork/Labelling | Artwork or labeling related flexibilities which may include any of the following:   1. Exemption of country specific details/artwork 2. Acceptance of Common English labels 3. Exemption of including Physical leaflets on product packs 4. Acceptance of QR Code on pack (to access local labels) in replacement of physical local labels |
| Administrative/reference documents | Administrative Documents related flexibilities which may include exemption and/or acceptance of alternate to any of the following:   1. Letter of Authorization (LOA) 2. Pricing Certificate/Price Details 3. Worldwide Registration Status 4. Declarations, Approval Letters (includes requirement for Legalization/Notarization of documents 5. Assessment Reports from reference agency |
| Other agilities | Administrative CTD-related flexibilities which may include exemption and/or acceptance of alternates to any of the following:   1. Provision of dossier/label in local Language, 2. Country specific dossier formatting requirements 3. Acceptance of rolling submission |
