# Supplementary material for: Regulatory agilities impacting review timelines for Pfizer/BioNTech’s BNT162b2 mRNA COVID-19 vaccine: a retrospective study
Source: Front Med (Lausanne). 2023 Nov 8;10:1275817. doi: 10.3389/fmed.2023.1275817 (PMC10664654; doi:10.3389/fmed.2023.1275817)
Supplement: Supplementary file 2 [file Table_2.DOCX]

Supplementary Table 2. Descriptive summary of review times and type of approval for COVID-19 vaccine granted for each country

| **Region** | **Country** | **Number of calendar days to approval** | **Type of Approval** |
| --- | --- | --- | --- |
| **Africa Middle East** | Algeria | 13 | EUA |
|  | Bahrain | 7 | EUA |
|  | Botswana | 0 | EUA |
|  | Egypt | 0 | EUA |
|  | eSwatini (Swaziland) | 0 | EUA |
|  | Ghana | 0 | EUA |
|  | Iran | 0 | EUA |
|  | Iraq | 13 | EUA |
|  | Jordan | 16 | EUA |
|  | Kenya | 0 | EUA |
|  | Kuwait | 17 | EUA |
|  | Lebanon | 12 | EUA |
|  | Mauritius | 0 | EUA |
|  | Morocco | 7 | EUA |
|  | Nigeria | 50 | EUA |
|  | Oman | 17 | EUA |
|  | Qatar | 14 | EUA |
|  | Rwanda | 0 | EUA |
|  | Saudi Arabia | 14 | EUA |
|  | South Africa | 35 | Import License |
|  | Tunisia | 33 | EUA |
|  | United Arab Emirates | 15 | EUA |
| **Others** | Australia | 93 | CMA |
|  | Canada | 98 | Other (please specify) |
|  | Japan | 58 | CMA |
|  | New Zealand | 105 | CMA |
| **Asia** | Bhutan | 0 | EUA |
|  | Brunei | 0 | EUA |
|  | Hong Kong | 21 | Import License |
|  | Indonesia | 27 | EUA |
|  | Macao | 21 | Import License |
|  | Malaysia | 25 | CMA |
|  | Maldives | 0 | EUA |
|  | Nepal | 12 | EUA |
|  | Pakistan | 14 | EUA |
|  | Philippines | 22 | EUA |
|  | Singapore | 10 | EUA |
|  | South Korea | 39 | CMA |
|  | Sri Lanka | 6 | EUA |
|  | Taiwan | 9 | EUA |
|  | Thailand | 14 | CMA |
|  | Vietnam | 11 | EUA |
| **Eurasia** | Albania | 9 | CMA |
|  | Armenia | 0 | EUA |
|  | Georgia | 0 | EUA |
|  | Israel | 10 | Full approval (MA) |
|  | Kazakhstan | 23 | Other (please specify) |
|  | Kosovo | 0 | CMA |
|  | Moldova | 0 | CMA |
|  | Mongolia | 0 | EUA |
|  | North Macedonia | 13 | CMA |
|  | Palestine | 0 | EUA |
|  | Serbia | 27 | EUA |
|  | Switzerland | 64 | CMA |
|  | Turkey | 10 | Import License |
|  | Ukraine | 6 | CMA |
| **European Economic Area** | EEA (+EU) = 30 | 21 | CMA |
| **Latin America** | Argentina | 20 | CMA |
|  | Brazil | 18 | CMA |
|  | Chile | 19 | Import License |
|  | Colombia | 5 | EUA |
|  | Costa Rica | 19 | EUA |
|  | Dominican Republic | 5 | EUA |
|  | Ecuador | 15 | Import License |
|  | El Salvador | 9 | EUA |
|  | Honduras | 2 | EUA |
|  | Mexico | 15 | EUA |
|  | Panama | 0 | EUA |
|  | Paraguay | 26 | EUA |
|  | Peru | 17 | CMA |
|  | Uruguay | 14 | EUA |
| **United Kingdom** | United Kingdom | 55 | EUA |
| **USA** | USA | 21 | EUA |
|  | USA | 97 | Biologics License Application |

EUA (Emergency Use Authorization)

CMA (Conditional Marketing Authorization)
